# Circulating miR-106b-3p, miR-101-3p and miR-1246 as diagnostic biomarkers of hepatocellular carcinoma

**DOI:** 10.18632/oncotarget.24601

**Published:** 2018-02-27

**Authors:** Farzaneh Moshiri, Alessandro Salvi, Laura Gramantieri, Angelo Sangiovanni, Paola Guerriero, Giuseppina De Petro, Cristian Bassi, Laura Lupini, Arash Sattari, Douglas Cheung, Dario Veneziano, Giovanni Nigita, Ram C. Shankaraiah, Nazario Portolani, Paolo Carcoforo, Francesca Fornari, Luigi Bolondi, Antonio Frassoldati, Silvia Sabbioni, Massimo Colombo, Carlo M. Croce, Massimo Negrini

**Affiliations:** ^1^ Department of Morphology, Surgery and Experimental Medicine, University of Ferrara, Ferrara, Italy; ^2^ Comprehensive Cancer Center, The Ohio State University, Columbus, Ohio, USA; ^3^ Department of Molecular and Translational Medicine, Division of Biology and Genetics, University of Brescia, Brescia, Italy; ^4^ Center for Applied Biomedical Research, St. Orsola-Malpighi University Hospital, Bologna, Italy; ^5^ Gastroenterology and Hepatology Division, Fondazione IRCCS Ca’ Granda Ospedale Maggiore Policlinico, University of Milano, Milan, Italy; ^6^ Department of Medical and Surgical Sciences, Surgical Clinic, University of Brescia, Brescia, Italy; ^7^ Department of Medical and Surgical Sciences, University of Bologna, Bologna, Italy; ^8^ Oncology Division, University Hospital of Ferrara, Cona (FE), Italy; ^9^ Department of Life Sciences and Biotechnology, University of Ferrara, Ferrara, Italy

**Keywords:** hepatocellular carcinoma, cirrhosis, circulating microRNA, diagnostic biomarkers

## Abstract

Hepatocellular carcinoma (HCC) is the most common liver cancer and second leading cause of cancer related death worldwide. Most HCCs occur in a damaged cirrhotic background and it may be difficult to discriminate between regenerative nodules and early HCCs. No dependable molecular biomarker exists for the early detection of HCC. MicroRNAs (miRNAs) have attracted attention as potential blood-based biomarkers. To identify circulating miRNAs with diagnostic potential in HCC, we performed preliminary RNAseq studies on plasma samples from a small set of HCC patients, cirrhotic patients and healthy controls. Then, out of the identified miRNAs, we investigated miR-101-3p, miR-106b-3p, miR-1246 and miR-411-5p in plasma of independent HCC patients’ cohorts. The use of droplet digital PCR (ddPCR) confirmed the aberrant levels of these miRNAs. The diagnostic performances of each miRNA and their combinations were measured using Receiver Operating Characteristic (ROC) curve analyses: a classifier consisting of miR-101-3p, miR-1246 and miR-106b-3p produced the best diagnostic precision in plasma of HCC vs. cirrhotic patients (AUC = 0.99). A similar performance was found when the levels of miRNAs of HCC patients were compared to healthy controls (AUC = 1.00). We extended the analyses of the same miRNAs to serum samples. In serum of HCC vs. cirrhotic patients, the combination of miR-101-3p and miR-106b-3p exhibited the best diagnostic accuracy with an AUC = 0.96. Thus, circulating miR-101-3p, miR-106b-3p and miR-1246, either individually or in combination, exhibit a considerable potential value as diagnostic biomarkers of HCC.

## INTRODUCTION

Hepatocellular carcinoma (HCC) ranks fifth in incidence and second for cancer mortality worldwide, with nearly 746,000 deaths in 2012 (according to the International Agency for Research on Cancer, IARC) [1]. Cirrhosis is present in 80–90% of HCC patients and represents the main risk factor for liver cancer [2]. In addition to hepatitis B and C viral infections and alcohol abuse as etiologic factors, cirrhosis may also develop in nonalcoholic fatty liver disease (NAFLD) and non-alcoholic steatohepatitis (NASH). NASH typically develops in a background of type 2 diabetes, dyslipidemia, hypertension and obesity, which have become important etiologic factors of HCC in the United States [3]. The last decades has witnessed a significant rise in the incidence of HCC in western countries [4] with annual increases of 3.6% and 2.9% in men and women, respectively.

The outcome of HCC is generally poor. Survival at 5 years is estimated 69% after curative surgery, which is only feasible in early stage HCC patients [5]. Survival drops to 3% in advanced HCCs [5], highlighting the need of diagnostic biomarkers for the early detection of HCC in at-risk subjects. Current diagnostic methods such as imaging techniques and serological tumor markers are far from optimal. In a variable percentage of cases, imaging techniques such as ultrasonography or computed tomography (CT) as well as histopathological examination on fine needle biopsies are inconclusive in the diagnostic characterization of small nodules in cirrhosis [6]. Accuracy of commonly used blood markers, alpha-fetoprotein (AFP) or des-gamma-carboxy prothrombin is modest, with false negative results in one-third of cases of HCC and a high rate of false positive results in patients with benign liver diseases, such as hepatitis and cirrhosis [7–9]. The American Association for the Study of Liver Diseases guidelines (AASLD) no longer recommends AFP to be used for diagnosis [10].

In the last few years, cell-free blood microRNAs (miRNAs) have received a growing interest as potential disease biomarkers. MiRNAs are 19–22 nucleotide-long non-coding RNAs that regulate gene expression mainly at the post-transcriptional level [11, 12]. Because of their altered expression in cancer cells, miRNAs have received attention for their fundamental role in controlling cancer-associated processes [13, 14]. The attention on miRNAs as potential biomarkers for cancer and other diseases was attracted because of their release and stability in bodily fluids, like blood, serum, plasma, urine etc. [15, 16]. Their presence inside microvesicles, exosomes and apoptotic bodies, or bound to RNA-binding proteins (e.g. AGO family members and High Density Lipoproteins), protects them from degradation while in circulation [17]. Therefore, the search for cancer-specific circulating miRNAs has been widely investigated [18, 19]. The search for an association of circulating miRNAs with HCC was also investigated [20–26]. The mainstream of these studies were often based on measuring miRNAs chosen on the basis of their deregulated expression in cancer tissues. However, comparison of results from different studies showed inconsistencies. Differences in results were common and possibly due to either variable miRNAs expression depending on etiology, stage, type of therapy or study design, sources and methods of sample collection, presence of contaminating cells, use of platforms with different sensitivity, which could all have been responsible for inconsistencies in the discovered patterns.

In an effort to assess the diagnostic performance of circulating miRNAs in HCC, we performed a multicenter study to search for HCC-associated miRNA signatures. Four miRNAs, which emerged in a preliminary RNAseq experiment, were validated using a droplet digital PCR (ddPCR) approach on independent cohorts of patients and controls.

## RESULTS

### Discovery and validation of microRNAs with differential levels in plasma of HCC patients versus control subjects

A case-control study was designed to identify miRNA profile able to discriminate HCC patients from cirrhotic or control population. The initial discovery phase, aimed at searching for differential circulating miRNAs in HCC patients vs. cirrhotic patients and vs. healthy control subjects, was performed on plasma RNAs from 4 HCC patients, 12 cirrhosis patients and 9 healthy individuals (not affected by any liver diseases or neoplastic disorder) using an Illumina HiSeq 2000 sequencing system.

In total, small RNA sequencing generated approximately 12 million of reads per sample. After removing low quality reads and adapter sequences from raw data, the remaining short reads were aligned to miRbase (release 21), using the STAR algorithm [27]. The abundance of each miRNA was counted using the HTSeq package and transformed according to the procedures described by Love and colleagues [28]. After selecting miRNAs with at least 1 read, 2496 different miRNAs were identified. For class comparison, we excluded miRNAs with less than 100 read counts on average and then performed a multiple class ANOVA test to compare the 3 classes of samples. Multiple class comparison revealed 38 differentially expressed miRNAs, which exhibited a fold change > 3 and a *p* value < 0.05 in at least one of the comparisons between HCC and cirrhosis or controls (Supplementary Table 1).

Among these, validation steps were performed on a subset of 9 miRNAs (miR-101-3p, miR-1246, miR-106b-3p, miR-411-5p, miR-3162-3p, miR-548at-5p, miR-5193, miR-1469 and miR-6726-5p). Validations were all performed by the use of droplet digital PCR (ddPCR) technology (Bio-Rad Laboratories Inc., CA, USA). ddPCR emerged as a robust method to quantify circulating miRNAs [29–31]; compared to real time PCR, ddPCR shows lower variations, thus it can better reach a statistically significant discrimination between cases and controls [30]. We employed Exiqon assays based on LNA PCR primers, as we have previously described [29, 31]. An initial technical validation was performed on plasma samples from 7 HCC patients, 10 cirrhosis patients and 7 controls, which overlapped with the samples employed for RNAseq analysis. Among selected miRNAs, four sets of primers were available as validated assays (miR-101-3p, miR-1246, miR-106b-3p, miR-411-5p), the remaining five sets were custom designed. Only the 4 validated assays produced interpretable results by ddPCR method, while custom assays produced positive droplets that could not be differentiated from background negative droplets.

The comparison between RNAseq and ddPCR methods indicated that both could measure the levels of all 4 circulating miRNAs in a consistent manner (Supplementary Figure 1). Both assays showed the same direction of dysregulation for all miRNAs. These data indicated that, when primer sets performed properly, ddPCR was a reliable and economical approach for quantifying circulating miRNAs.

### microRNAs with differential levels in plasma of HCC patients versus control subjects

We evaluated the 4 candidate miRNAs (miR-101-3p, miR-1246, miR-106b-3p, miR-411-5p), which emerged in a preliminary RNAseq experiment, in independent cohorts of HCC patients, in patients with liver cirrhosis without cancer, and in healthy individuals.

In addition to the above mentioned cohort 1, which included 7 HCC patients, 10 cirrhosis patients and 7 controls (from the University Hospital of Milan), two additional independent cohorts were analyzed (Table 1): cohort 2, plasmas from 9 HCC and 6 cirrhotic patients (from the University Hospital of Ferrara); cohort 3, plasmas from 22 HCC patients and 11 healthy controls (from the University Hospital of Brescia).

**Table 1:** Characteristics of study participants

			Cohort 1 (Plasma)			Cohort 2 (Plasma)		Cohort 3 (Plasma)		Cohort 4 (Serum)	
			HCC	Cirrhosis	Control	HCC	Cirrhosis	HCC	Control	HCC	Cirrhosis
			(*N* = 7)	(*N* = 21)	(*N* = 14)	(*N* = 9)	(*N* = 6)	(*N* = 22)	(*N* = 11)	(*N* = 24)	(*N* = 14)
Age (Years)	< 60	N (%) (Range)	1 (14.3%) (57)	6 (28.6%) (41–59)	6 (42.8%) (29–44)	0 (0%)	1 (16.7%) (57)	2 (9.1%) (48–54)	3 (27.3%) (50–57)	4 (16.7%) (47–56)	5 (35.7%) (32–54)
	≥ 60	N (%) (Range)	6 (85.7%) (60–83)	15 (71.4%) (60–79)	8 (57.2%) (53–77)	9 (100%) (61–71)	5 (83.3%) (61–71)	20 (90.9%) (61–71)	8 (72.7%) (61–74)	20 (83.3%) (62–77)	9 (64.3%) (61–70)
Gender	Male	N (%)	5 (71.4%)	13 (61.9%)	6 (42.8%)	7 (77.8%)	5 (83.3%)	16 (72.7%)	8 (72.7%)	19 (79.2%)	12 (85.7%)
	Female	N (%)	2 (28.6%)	8 (38.1%)	8 (57.2%)	2 (22.2%)	1 (16.7%)	6 (27.3%)	3 (27.3%)	5 (20.8%)	2 (14.3%)
	HBV	N (%)	2 (28.6%)	7 (33.3%)		0 (0%)		7 (31.8%)		----	
	HCV	N (%)	5 (71.4%)	13 (61.9%)		6 (66.7%)		9 (40.9%)		14 (58.3%)	
Etiology	Mixed Infection	N (%)	0 (0%)	0 (0%)		0 (0%)		2 (9.1%)		1 (4.2%)	
	ETOH	N (%)	0 (0%)	1 (4.8%)		0 (0%)		0 (0%)		3 (12.5%)	
	Others	N (%)	0 (0%)	0 (0%)		0 (0%)		0 (0%)		3 (12.5%)	
	Unknown	N (%)	0 (0%)	0 (0%)		3 (33.3%)		4 (18.2%)		3 (12.5%)	
AFP (ng/μl)	< 20	N (%)	3 (42.9%)	18 (85.7%)		5 (55.6%)		6 (27.3%)		18 (75.0%)	
	20–400	N (%)	3 (42.9%)	3 (14.3%)		4 (44.4%)		12 (54.5%)		6 (25%)	
	> 400	N (%)	1 (14.3%)	0 (0%)		0 (0%)		4 (18.2%)		0 (0%)	
Child-Pugh	A		5	21		9	6			24	14
	B		1	0		0	0			0	0
	C		1	0		0	0			0	0
Nodules Size (cm)	< 3	N (%) (Range)	5 (71.4%) (1–2.8)					8 (36.4%) (0.4–2.8)		21 (87.5%) (0.8–2.9)	
	≥ 3	N (%) (Range)	2 (28.6%) (3)					14 (63.6%) (3–9)		3 (12.5%) (3–4.5)	

**Figure 1:**
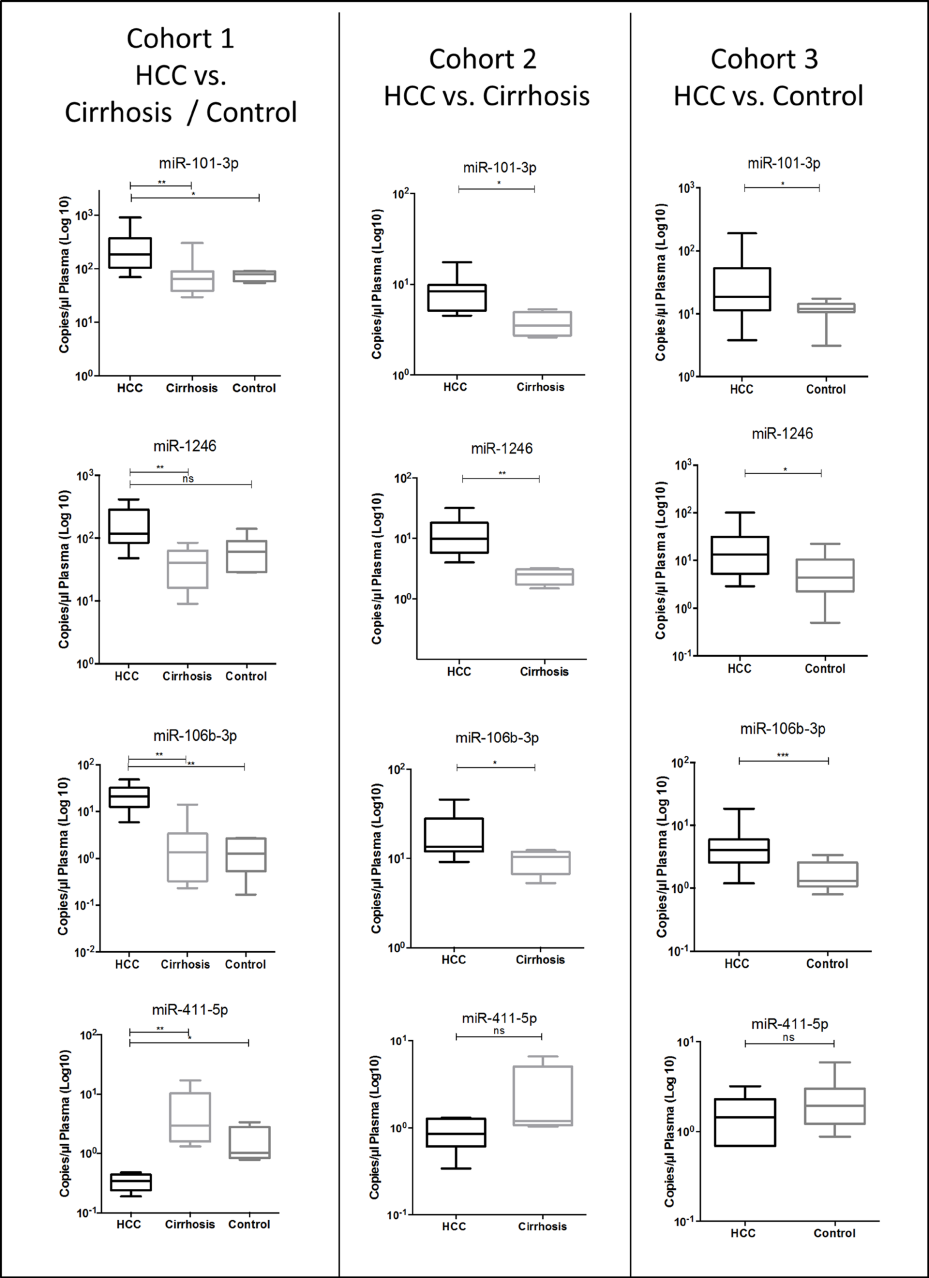
Validation of differential levels of plasma miR-101-3p, miR-1246, miR-106b-3p and miR-411-5p using droplet digital PCR absolute quantification in various cohorts of HCC vs. cirrhosis and HCC vs. healthy control Mann-Whitney *U* test; ^*^p value < 0.05, ^**^p value < 0.005, ^***^p value < 0.001, ns: *p* value ≥ 0.05.

In all cohorts, the levels of circulating miR-101-3p, miR-1246 and miR-106b-3p were significantly up-regulated in HCC patients compared to controls, while the differential level of miR-411-5p did reach significance only in cohort 1 (Figure 1).

ddPCR is a method that can measure the absolute concentration of the investigated nucleic acid. In these analyses, while there is concordance in the qualitative trend of results, there are differences in absolute levels of miRNAs measured in the different sets of samples. This is justified and partly expected, as samples were subjected to different pre-analytical steps: not only they were collected at different institutions at different time, but also purified starting from different amount of plasma using different methods.

### Diagnostic performance of circulating miRNAs exhibiting differential levels in HCC patients versus controls

**Figure 2:**
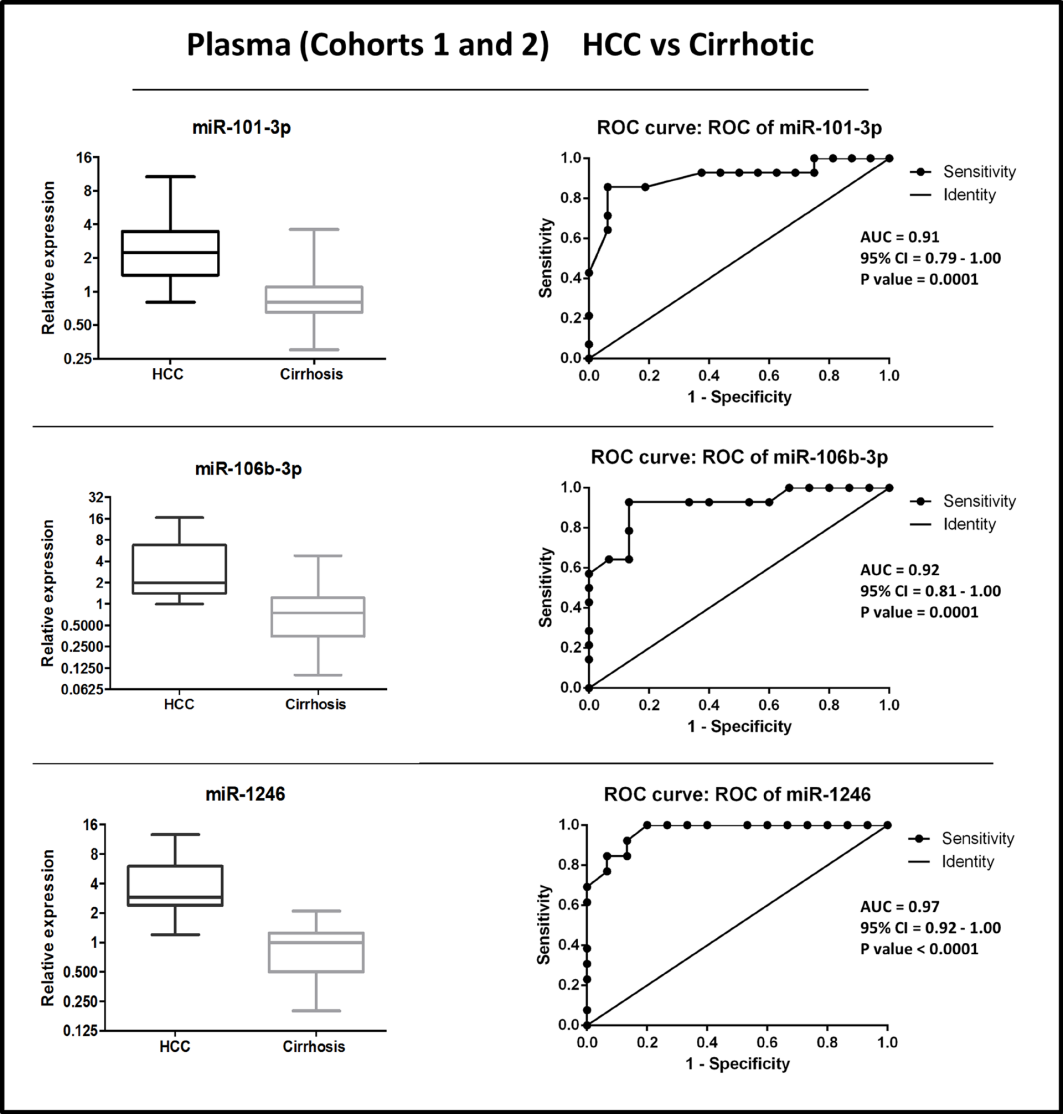
Distribution of levels and ROC curve analysis of plasma miR-101-3p, miR-1246, miR-106b-3p in combined cohorts (1 + 2) of HCC vs. cirrhosis AUC: Area Under the ROC Curve. CI: Confidence Interval.

**Figure 3:**
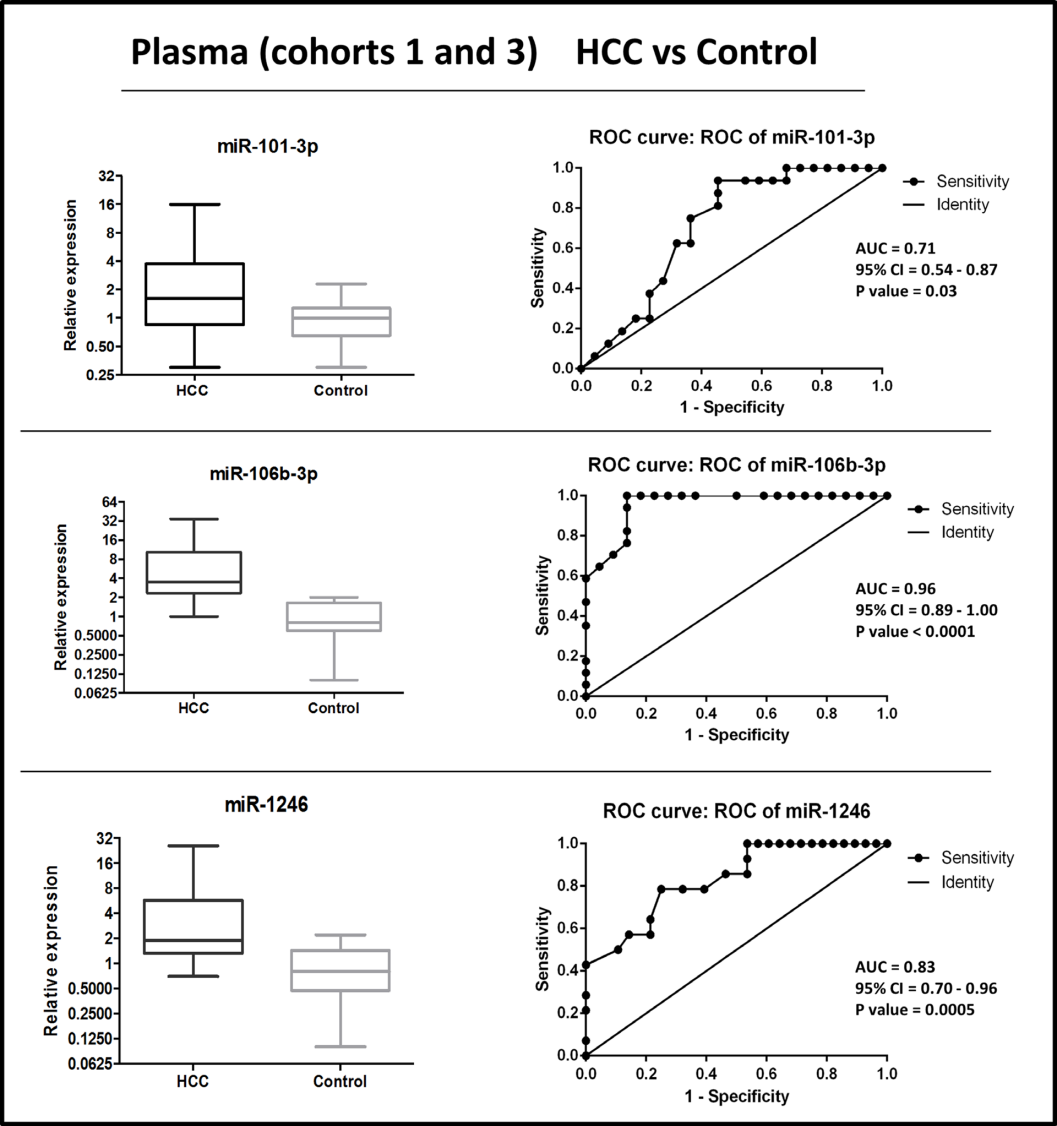
Distribution of levels and ROC curve analysis of plasma miR-101-3p, miR-1246, miR-106b-3p in combined cohorts (1 + 3) of HCC vs. healthy control AUC: Area Under the ROC Curve. CI: Confidence Interval.

We assessed the diagnostic performance of plasma miRNAs that exhibited differential levels between HCC patients and controls. Receiver Operating Characteristic (ROC) analyses were performed on the combined cohorts 1 + 2 (plasma from HCC versus cirrhotic patients) and on cohorts 1 + 3 (plasma from HCC vs healthy controls). To combine data from different cohorts, values of each set were normalized on the mean copies/ul value from non-cancer individuals (cirrhotic or healthy control). Normalized data distribution and individual ROCs are shown in Figures 2–3.

To establish classifiers made of miRNA combinations, logistic regression models were generated in Weka software 3.8.0 (the University of Waikato, Hamilton, New Zealand) using miRNAs normalized data (Supplementary Table 2).

**Figure 4:**
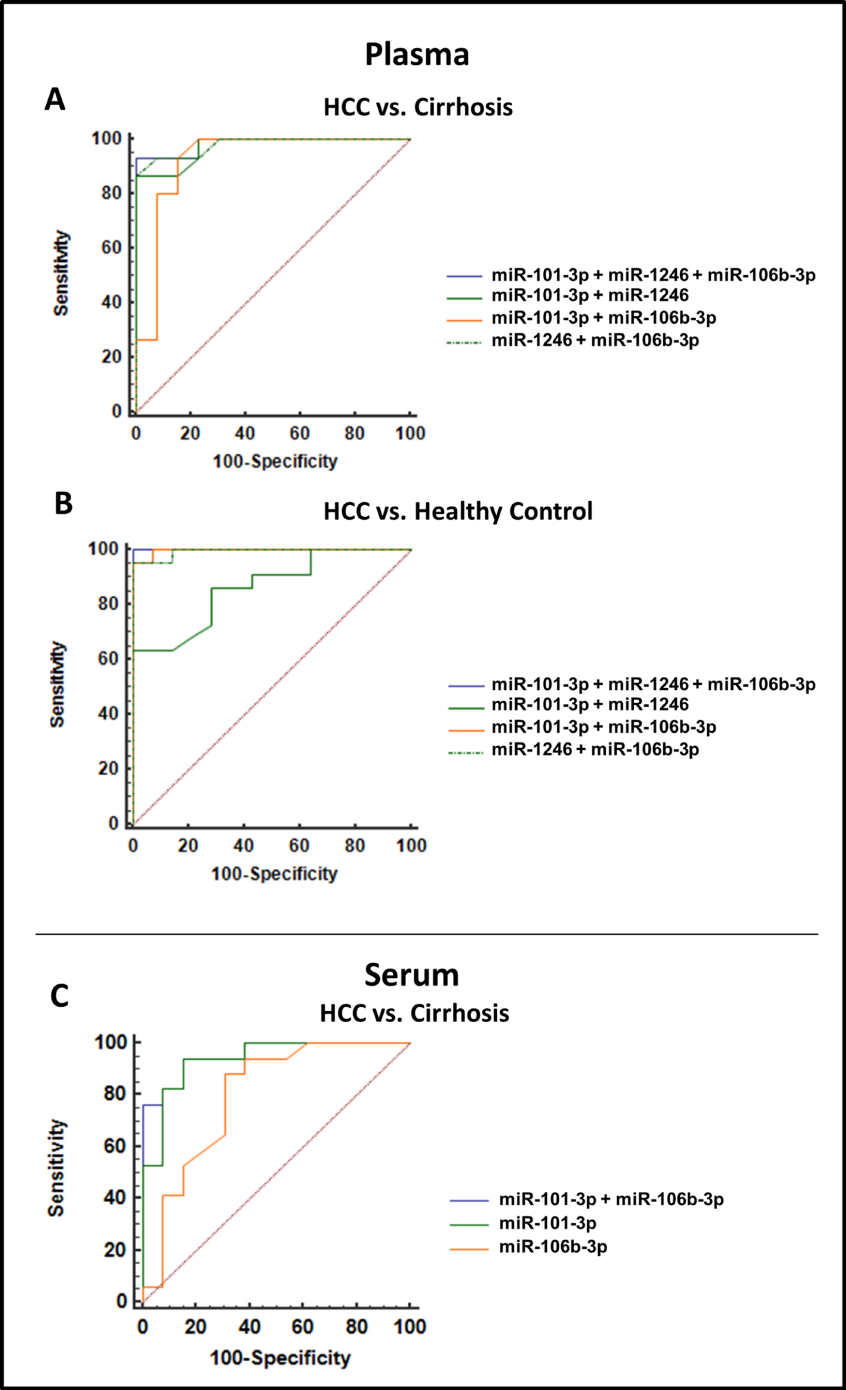
Comparison of ROC Curves display the diagnostic precision of miRNA classifiers (**A**) Plasma miRNA classifiers HCC vs. cirrhosis. (**B**) Plasma miRNA classifiers HCC vs. healthy control. (**C**) Serum miRNA classifier HCC vs. cirrhosis.

Results from each classifier were used to evaluate the diagnostic performances, calculated by ROC analyses. Results for multi miRNA classifiers indicated that all combinations had superior diagnostic accuracy than single ones (Table 2 and Figure 4A–4B).

**Table 2:** Diagnostic performance of plasma/serum miRNAs in HCC vs. cirrhosis patients or healthy controls

miRNAs	Experimental condition	Test Fluid	Sensitivity	Specificity	Accuracy	AUC	SE	95% CI	*p* value
miR-101-3p	HCC vs. cirrhosis	Plasma	86.7	80.0	83.3	0.91	0.06	0.743 to 0.982	< 1.00E-04
miR-1246	HCC vs. cirrhosis	Plasma	86.7	84.6	85.7	0.97	0.02	0.824 to 1.000	< 1.00E-04
miR-106b-3p	HCC vs. cirrhosis	Plasma	90.9	72.2	79.3	0.91	0.05	0.750 to 0.986	< 1.00E-04
miR-101-3p + miR-106b-3p	HCC vs. cirrhosis	Plasma	85.7	78.6	82.1	0.93	0.06	0.761 to 0.990	< 1.00E-04
miR-101-3p + miR-1246	HCC vs. cirrhosis	Plasma	92.9	85.7	89.3	0.97	0.02	0.824 to 1.000	< 1.00E-04
miR-106b-3p + miR-1246	HCC vs. cirrhosis	Plasma	87.5	91.7	89.3	0.98	0.02	0.841 to 1.000	< 1.00E-04
miR-101-3p + miR-106b-3p + miR-1246	HCC vs. cirrhosis	Plasma	100.0	92.9	96.4	0.99	0.02	0.849 to 1000	< 1.00E-04
miR-101-3p	HCC vs. Control	Plasma	71.4	58.8	65.8	0.71	0.08	0.538 to 0.843	1.00E-02
miR-1246	HCC vs. Control	Plasma	57.1	78.6	71.4	0.83	0.06	0.684 to 0.929	< 1.00E-04
miR-106b-3p	HCC vs. Control	Plasma	87.0	83.3	85.4	0.95	0.03	0.837 to 0.995	< 1.00E-04
miR-101-3p + miR-106b-3p	HCC vs. Control	Plasma	95.7	92.9	94.4	1.00	0.01	0.896 to 1.000	< 1.00E-04
miR-101-3p + miR-1246	HCC vs. Control	Plasma	81.8	71.4	77.8	0.86	0.06	0.708 to 0.955	< 1.00E-04
miR-106b-3p + miR-1246	HCC vs. Control	Plasma	91.3	92.3	91.7	0.99	0.01	0.890 to 1.000	< 1.00E-04
miR-101-3p + miR-106b-3p + miR-1246	HCC vs. Control	Plasma	100.0	100.0	100.0	1.00	0.00	0.903 to 1.000	< 1.00E-04
miR-101-3p	HCC vs. cirrhosis	Serum	84.6	94.1	90.0	0.94	0.04	0.784 to 0.993	< 1.00E-04
miR-106b-3p	HCC vs. cirrhosis	Serum	69.2	76.5	73.3	0.80	0.08	0.613 to 0.922	6.00E-03
miR-101-3p + miR-106b-3p	HCC vs. cirrhosis	Serum	84.6	94.1	90.0	0.96	0.03	0.823 to 0.999	< 1.00E-04

Sensitivity: percent of correctly classified HCC patients.

Specificity: percent of correctly classified non-HCC subjects.

Accuracy: Percent of cases correctly classified.

AUC: Area Under the Curve.

SE: Standard Error.

CI: Confidence Interval.

### Analysis of differential microRNAs in serum samples

**Figure 5:**
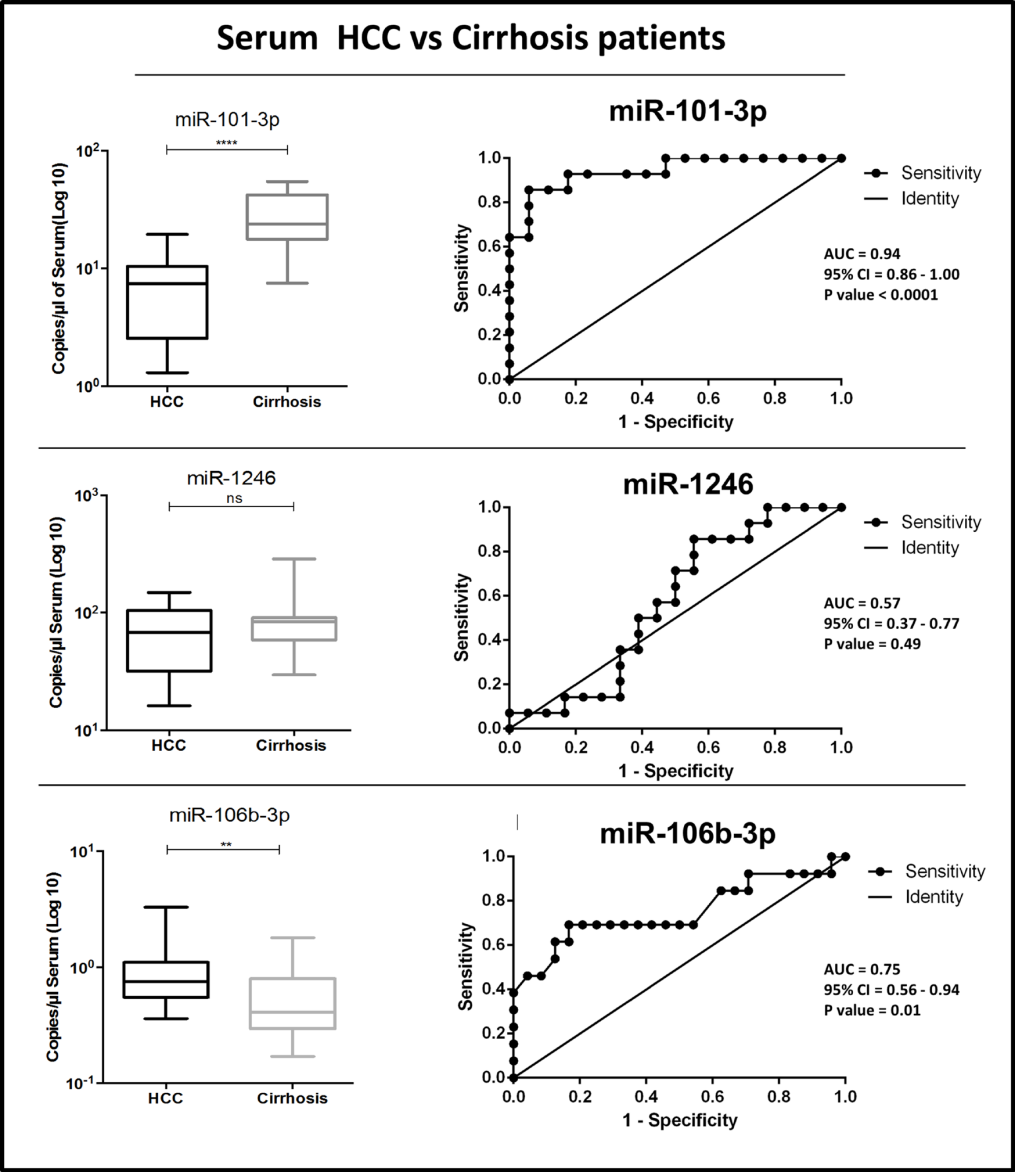
Distribution of levels and ROC curve analysis of serum miRNAs miR-101-3p, miR-1246 and miR-106b-3p, in HCC vs. cirrhosis patients AUC: Area Under the ROC Curve. CI: Confidence Interval.

Given the known variability in spectrum and amount of circulating miRNAs in plasma compared to serum [32], we evaluated the 4 selected candidate miRNAs (miR-101-3p, miR-1246, miR-106b-3p, miR-411-5p) also in serum samples of an independent cohort of HCC and cirrhotic samples (cohort 4, Table 1). Serum samples from 24 HCC patients and 14 cirrhosis patients were used. The results were in some way contrasting: while no different concentrations of miR-1246 and miR-411-5p were detected in HCC vs. cirrhotic patients, miR-106b-3p retained its significant higher level in HCC patients, while miR-101-3p was instead significantly down-regulated in HCC patients (Figure 5). ROC analyses were also performed (Figure 5). In serum samples, miR-101-3p and miR-106b-3p exhibited good diagnostic accuracies. By generating a logistic model (= −2.4903 −0.2632 copies/μl miR-101-3p + 2.093 x copies/μl miR-106b-3p) for results of serum samples, we assessed the diagnostic performance of combined miR-101-3p and miR-106b-3p (Table 2). ROC analysis (Figure 4C) showed that the combination of serum miR-101-3p and miR-106b-3p could discriminate HCC from cirrhotic patients with AUC = 0.96, sensitivity 84.6%, specificity 94.1% and accuracy 90.0%.

### miR-122 level is plasma and serum of HCC patients

**Figure 6:**
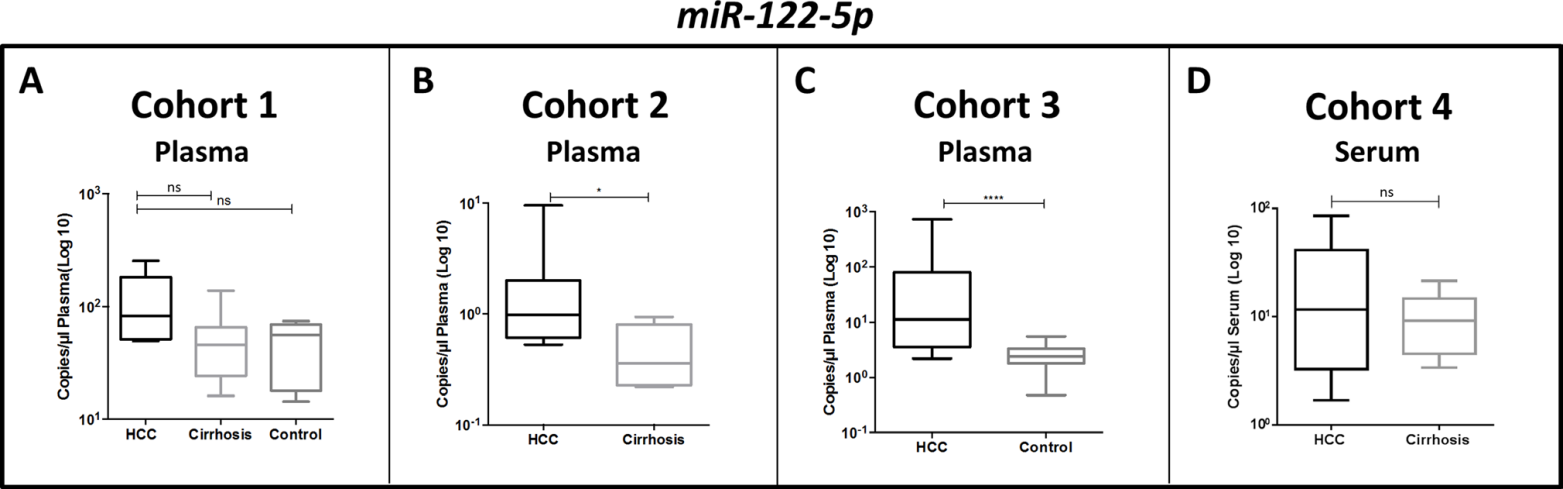
Differential levels of miR-122-5p in HCC patients versus controls (cirrhosis patients or healthy controls) in plasma and serum samples Mann-Whitney *U* test; ^*^p value < 0.05, ^****^p value < 0.0001, ns *p* value ≥ 0.05.

Since damaged liver may release liver miRNAs in circulation, we also investigated the level of miR-122-5p, the most abundant liver-specific miRNA, in plasma and serum of HCC patients in comparison with cirrhotic patients or healthy individuals. In plasma samples, miR-122-5p revealed a higher concentration in HCC vs. controls (Figure 6A–6C), with the most significant difference being observed between HCC patients and healthy controls of cohort 3. Serum samples showed no significant differences between HCC and cirrhotic patients (Figure 6D).

### Association of circulating candidate miRNAs with clinico-pathological features of HCC patients

We examined the correlation between circulating miR-101-3p, miR-1246 and miR-106b-3p levels with clinic-pathological factors in HCC patients of cohort 3 (the largest cohort with complete data). By using the mean value of plasma (copies/μl) as cut off threshold, we calculated the association of each miRNA to available features in HCC patients. No correlation was found between plasma miRNA levels and patients age, gender, viral status, AFP level, grade, number and size of tumor (Supplementary Table 3).

## DISCUSSION

After an initial RNAseq discovery phase performed on a small number of plasma samples, 4 miRNAs were further analyzed in independent cohorts of patients and controls to evaluate their significance as diagnostic circulating biomarkers using a ddPCR approach [33]. ddPCR emerged as a robust method to quantify circulating miRNAs [29–31]; compared to real time PCR, ddPCR shows lower variations, thus it can better reach a statistically significant discrimination between cases and controls [30].

A significantly higher level of miR-101-3p, miR-1246 and miR-106b-3p was found in the plasma of HCC patients in comparison with control subjects, irrespective of the fact that controls were cirrhotic or healthy individuals. Each one of these miRNAs exhibited an excellent diagnostic accuracy, as shown by individual ROCs. When combined, their diagnostic performance further improved to reach a maximum of AUC = 0.99 for HCC vs cirrhotic patients and AUC = 1.00 for HCC vs healthy subjects. Starting from small cohorts of different sources poses some risk of not reaching consistent or significant results. In this study, instead, results from the various cohorts were coherent, thereby supporting their consistency.

The same miRNAs were also analyzed in serum samples, another common source of circulating miRNAs. Two of the miRNAs, miR-106b-3p and miR-101-3p, retained their significant deregulation in HCC compared to cirrhotic patients, but miR-101-3p was strongly down-regulated instead of being up-regulated.

Albeit early studies reported that composition and levels of miRNAs in serum and plasma are similar [15], there are several examples that contradict this idea. Heegaard and colleagues [34] tested miRNA levels in paired serum and plasma samples of non-small cell lung cancer patients. From the analysis of 30 miRNAs, they concluded that serum and plasma might be very different in miRNA levels. Examples exist also in liver cancer studies: two reports found miR-223-3p consistently low in plasma of HCC patients [35, 36], and two other studies found the same miRNA high in the serum of HCC patients [37, 38]. Two reports indicate that miR-21 low in serum of HCC patients [37, 39], but other studies reported that miR-21 is high in plasma of HBV-related HCC patients [40]. Although these results are puzzling, we should conclude that differences between plasma and serum are common and not unique finding of the present study.

At present, we can only speculate for possible biological reasons. It is believed that miRNAs are stable in circulation because protected by their inclusion into microvesicles and exosomes or by binding to RNA-binding proteins, like Argonaut, or lipoproteins. Serum samples originate from clotted whole blood. It is conceivable that during coagulation, the content of platelets, which includes miRNAs, is released in the microenvironment, where, because of the simultaneous release of several enzymes, they are possibly rapidly degraded. Conversely, plasma is obtained by centrifugation of non-coagulated whole blood. When plasma is prepared, platelets as well as other small vesicles are retained in the fluids and microRNAs protected from degradation. Thus, miRNAs isolated from plasma contain platelet-derived miRNAs, which may represent an important portion of the measured miRNAs. On the other hand, this pre-analytical step may also represent a source of variability, as the contribution of plateled-derived miRNAs to the composition of circulating miRNAs may vary depending on the applied centrifugal force during plasma preparation. The above reasoning may justify the common finding that most miRNAs are more abundant in plasma than in serum, and may also explain differences between levels of serum and plasma miRNAs as well as the many variable results reported in the literature [41].

Another apparently contradictory result is the finding of circulating miRNAs with an opposite regulation in tumors: for example miR-122 or miR-101-3p are upregulated in plasma, but downregulated in HCC [42–45], or the mentioned miR-21 downregulated in serum, but upregulated in HCC tissue [45, 46]. These are just a few examples of studies on circulating miRNAs, whose regulation was different from what found in tumor tissues. These findings could potentially raise doubts about the function of circulating miRNAs as truly tumor biomarkers. Considering that cancer-specific DNA in circulation can be less than 0.1% for non-metastatic tumors and 1–10% for large metastatic tumors, it seems conceivable that cancer-specific miRNAs might also represent just a minor fraction of all miRNAs in circulation. The question that arise is: can the profile of circulating miRNAs reflect the presence of a cancer, even if they do not originate from tumor cells,? Indeed, it is known that systemic pathophysiologic processes can be influenced as well as affect the development of cancer. The recent review by McAllister and Weinberg [47] highlights the evidences in support of the view of cancer as a systemic disease, with the involvement of organs and tissues distant from the primary tumor site. Thus, it seems likely that tumor-associated systemic processes may also affect the release of miRNAs from non-tumor cells. Thus, the release of miRNAs that do not originate from cancer cells may be indirectly influenced by the presence of a tumor and types and abundance of circulating miRNAs may not correlate with cancer-specific tissue miRNAs.

Among the investigated miRNAs, miR-101-3p was the one with the most striking difference between plasma and serum. The down-regulation of miR-101-3p has been previously reported in serum samples of HCC patients [22, 48]. In the study by Xie et al. [48], the capability of miR-101 to classify HCC and cirrhosis patients was associated with an AUC = 0.97 (95.5% sensitivity and 90.2% specificity), results that are very similar to ours. Instead, the upregulation of miR-101-3p in plasma of HCC patients has not been previously reported. miR-101 is known to be down-regulated in HCCs where it can act as a tumor suppressor miRNA [42, 43, 49].

High levels of plasma miR-1246 has been recently reported to be a biomarker for acute hepatic injury, a predictor of HCC recurrence and of overall and disease-free survival in HCC patients after liver transplantation [50]. Our study expands the potential clinical usefulness of miR-1246 as a plasma, but not serum, diagnostic biomarker of HCC. Very recently, Chai and colleagues found that miR-1246 is elevated in plasma samples and represent an extremely sensitive and specific biomarker for HCC diagnosis, with an area under curve (AUC) of 0.982 [51], results very similar to ours. They also report that miR-1246 can promote cancer self-renewal, drug resistance, tumorigenicity, and metastasis [51].

We also report that higher levels of circulating miR-106b-3p are present in HCC patients in comparison with either cirrhotic or healthy control subjects. Interestingly, among all validated miRNAs, miR-106b-3p was the only one to show an up-regulation both in plasma and serum samples. These results are in agreement with recent reports [52, 53]. In HCC, miR-106b and miRNAs of the miR-106b-25 cluster are upregulated, promote cell migration and metastasis and are associated with poor prognosis [54–56].

As individual biomarkers, all the three miRNAs had a very good diagnostic potential, as shown by ROC analyses, in all the cases superior to AFP (see for example the AFP ROC analysis, AUC = 0.79, shown in [22]). When miRNAs were combined, the plasma 3-miRNA classifier or the serum 2-miRNA classifier exhibited very strong diagnostic performances.

Although not identified in preliminary RNAseq analyses, we also investigated circulating miR-122-5p, a liver specific miRNA, whose up-regulation was detected in a number of studies, mostly correlated with liver injury, chronic hepatitis, non-alcoholic fatty-liver disease and cirrhotic patients compared to healthy population [37, 38, 57–60]. Thus, we used this miRNA as a reliable control to compare our results with those from other published reports on circulating miRNAs in HCC patients. Qi, et al. [37] reported an increased level of serum miR-122-5p in HBV-related HCC patients, but they also detected an increase in the sera of HBV patients without HCC compared to healthy controls. Similarly, El-Garem and colleagues reported no significant differences in serum miR-122-5p between HCC and non-HCC controls (chronic hepatitis and cirrhosis), while an increase of miR-122 was observed in HCC or cirrhotic patients compared to healthy controls [60]. These results suggest that high circulating miR-122-5p reflects liver damage rather than the presence of an underlying HCC. Our results are in agreement with this conclusion: we detected the most appreciable up-regulation of miR-122-5p in plasma of HCC patients in comparison with liver-healthy controls. Instead, when HCC and cirrhotic patients were compared, only a weak up-regulation was found in plasma and no significant difference was found in serum.

In conclusion, based on multiple independent cohorts and two different technological approaches, this study points to the diagnostic potential of three circulating miRNAs, miR-101-3p, miR-1246 and miR-106b-3p, to discriminate HCC patients from different control populations. Albeit a controlled clinical investigation is needed for validation, this study provides the indications that are necessary for the design of a clinical study. Of particular clinical value are the differences observed between HCC and cirrhotic patients, suggesting the potential usefulness of these circulating miRNAs as biomarkers for the early detection of HCC in this group of high-risk patients. The availability of noninvasive biomarkers that can be assayed over time during the monitoring of small liver nodules, might add relevant information to tailor follow-up and surveillance programs.

## MATERIALS AND METHODS

### Study design and subjects

Overall, the present study enrolled 128 participants, which included 62 clinically diagnosed HCC patients, 41 patients with liver cirrhosis and 25 healthy controls (not affected by liver diseases or any neoplastic disorder) from various Institutions, between April 2012 and November 2015. Cirrhosis was defined according to Child-Pugh-Turcotte clinical criteria. Demographic characteristics and clinical features of participants are shown in Table 1. Samples from HCC patients were collected prior to any therapeutic intervention. Samples from cirrhotic patients were collected during periodic follow-up visits and the patient exhibited no clinical evidence of cancer. Healthy control subjects were either healthy blood donors or were recruited prior to hernia surgery and had no evidence of liver dysfunction based on clinical reports. The study was approved by the local Ethic Committees of the University Hospitals of Milan, Ferrara, Brescia and Bologna; Italy, where the samples were collected. All participants provided written informed consent for the use of their samples for research purposes.

### Plasma and serum preparation

For plasma preparation, 5 ml of blood were collected in VACUETTE^®^ blood collection tubes containing EDTA as anti-coagulant. Blood was centrifuged twice at 1500 × g for 15 min. The cell free plasma was transferred to a new tube and stored at −80°C. For serum preparation, 5 ml of blood was drawn into a VACUETTE^®^ tube without anticoagulant. After clotting in standing position for 60 min at room temperature, the samples were centrifuged at 1000 × g for 10 min; the supernatant was quickly removed and stored at −80°C. Samples showing hemolysis were excluded.

### RNA isolation

For cohort 1, total RNA was isolated from 500 μl of plasma using Total RNA Purification Kit (Cat.17200, Norgen, Biotek Corp. Thorold, ON, Canada) according to manufacturer’s protocol. For cohorts 2, 3 and 4, total RNA was isolated from 200 μl of plasma or serum using miRNeasy Mini Kit (Cat.217004, Qiagen, Hilden, Germany) according to manufacturer’s protocol.

### Reverse transcription and droplet digital PCR

The candidate miRNAs acquired on next generation sequencing platforms, trained and further validated using QX200™ Droplet Digital™ PCR System (Bio-Rad Laboratories Inc., CA, USA), which provides absolute quantification of target molecules. 3 μl purified RNA was reverse transcribed in a 20 μl reaction using the miRCURY Locked Nucleic Acid (LNATM) Universal Reverse Transcription (RT) miRNA PCR, Polyadenylation and cDNA synthesis kit (Exiqon Inc., Vedbaek, Denmark). cDNA was diluted 50 × and PCR was performed according to the QX200 EvaGreen ddPCR protocol; In brief, 8 μl diluted cDNA, 10 μl 2X EvaGreen supermix (Bio-Rad) and appropriate amount of miRCURY LNA PCR primer sets (Exiqon) for each miRNA was amplified individually in a final volume of 20 μl. Each miRNA assayed once. LNA primers were used as follow: 1 μl at 56°C for miR-101-3p, miR-106b-3p and miR-411-5p and 1 μl at 58°C for miR-1246 and miR-122-5p. Droplet digital PCR was performed as described [29, 31].

### Statistical analysis

Differential expressions of individual miRNAs were analyzed in GraphPad Prism 5.01 (GraphPad software, CA, USA) software. The non-parametric Mann-Whitney *U*-test was used to calculate the significance of differences in circulating miRNA levels among comparing groups, as miRNA expression data were not normally distributed. Diagnostic performance of circulating miRNAs to discriminate HCC patients was evaluated using Receiver Operating Characteristic (ROC) curve analysis [61, 62]. To assess the diagnostic values of multi-miRNAs assays, logistic regression method [63] was applied. Logistic regression model was created in Weka 3.8.0 (The University of Waikato, Hamilton, New Zealand) software. The value of each sample was resolved by entering normalized data of miRNAs into the logistic model. The diagnostic performance of combinations classifiers as well as individual miRNAs was computed using MedCalc 16.4.3 (MedCalc, Ostend, Belgium). Contingency of clinic-pathological features in HCCs and miRNA expression were analyzed by the use of Fisher’s exact test. Two-tailed *p*-value was calculated and considered statistically significant at *p* < 0.05.

## SUPPLEMENTARY MATERIALS FIGURE AND TABLES



## Abbreviations

HCC: Hepatocellular Carcinoma
miRNA: microRNA
ddPCR: Droplet digital PCR
ROC: Receiver Operating Characteristic
AUC: Area Under the Curve
AFP: alpha feto protein
