# Supplementary material for: Circulating miR-106b-3p, miR-101-3p and miR-1246 as diagnostic biomarkers of hepatocellular carcinoma
Source: Oncotarget. 2018 Feb 27;9(20):15350–64. doi: 10.18632/oncotarget.24601 (PMC5880609; doi:10.18632/oncotarget.24601)
Supplement: Supplementary file 1 [file oncotarget-09-15350-s001.pdf]

Circulating miR-106b-3p, miR-101-3p and miR-1246 as diagnostic biomarkers of hepatocellular carcinoma

SUPPLEMENTARY MATERIALS

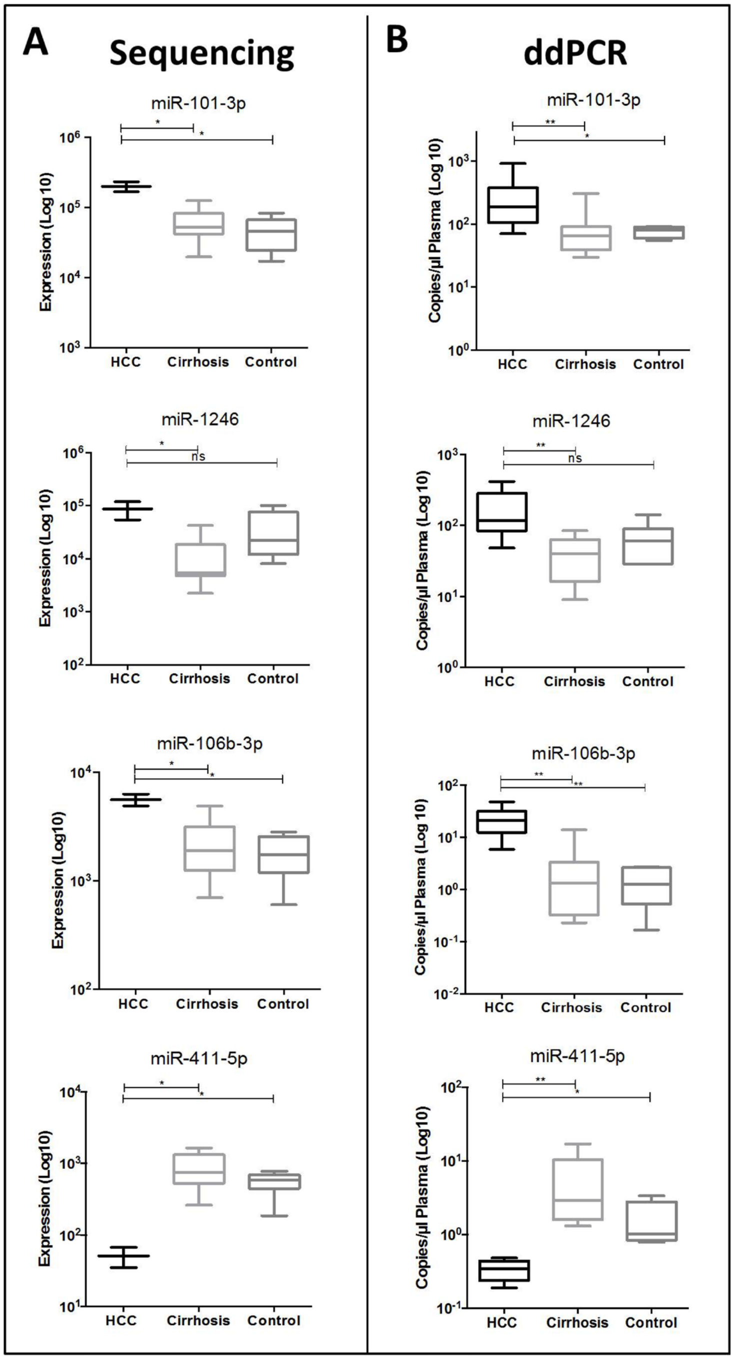

Supplementary Figure 1: Differential expression of plasma miRNAs in HCC patients compared to cirrhotic as well as healthy control participants, enrolled for cohort 1. The boxes represent the 25%–75% percentiles; the lines inside the box represent the median and the upper and lower lines representing the 10%–90% percentiles of fold change in plasma circulating levels of assayed miRNAs. A. Illumina sequencing normalized data. B. Droplet Digital PCR (ddPCR) absolute quantification. Mann-Whitney *U* test; $^{*}p$ value < 0.05, $^{**}p$ value < 0.005, ns: p value ≥ 0.05.

Supplementary Table 1: Differential levels of plasma miRNAs in HCC vs. cirrhosis and HCC vs. healthy controls from small RNA sequencing

	HCC vs. Cirrhosis			HCC vs. Healthy		
**miRNA ID**	**Corrected *p*-value**	**Absolute Fold change**	**Regulation**	**Corrected *p*-value**	**Absolute Fold change**	**Regulation**
hsa-miR-411-5p	4.80E-02	11.47	Down	1.16E-02	8.41	Down
hsa-miR-4446-3p	4.80E-02	17.33	Down	2.34E-01	3.46	Down
hsa-miR-6082	4.28E-02	5.58	Down	3.72E-01	2.62	Down
hsa-miR-665	5.76E-07	6.77	Up	9.36E-03	2.8	Up
hsa-miR-1469	3.22E-06	30.47	Up	2.11E-03	5.64	Up
hsa-miR-6726-5p	6.41E-05	19.74	Up	2.67E-01	2.85	Up
hsa-miR-3177-3p	2.37E-04	4.28	Up	5.21E-02	3.48	Up
hsa-miR-663b	5.35E-04	5.79	Up	7.47E-01	1.2	Up
hsa-miR-6073	6.41E-04	3.07	Up	2.32E-02	1.94	Up
hsa-miR-4713-5p	1.38E-03	5.63	Up	4.89E-01	1.53	Up
hsa-miR-3162-3p	1.63E-03	11.53	Up	4.19E-02	3.95	Up
hsa-miR-3621	1.63E-03	4.95	Up	4.19E-02	2.95	Up
hsa-miR-4741	1.63E-03	8.2	Up	2.00E-01	2.91	Up
hsa-miR-5702	1.63E-03	5.91	Up	4.19E-02	4.32	Up
hsa-miR-101-3p	1.86E-03	3.17	Up	1.02E-03	4.27	Up
hsa-miR-548f-5p	2.05E-03	3.75	Up	9.24E-05	5.82	Up
hsa-miR-1246	2.09E-03	6.58	Up	2.36E-01	2.2	Up
hsa-miR-6892-5p	3.07E-03	4.92	Up	9.11E-02	2.56	Up
hsa-miR-8073	3.07E-03	7.57	Up	1.54E-01	2.46	Up
hsa-miR-4516	3.65E-03	3.55	Up	2.53E-01	1.91	Up
hsa-miR-1228-5p	4.93E-03	4.34	Up	1.40E-01	2.32	Up
hsa-miR-548at-5p	4.99E-03	33.22	Up	4.76E-02	10.37	Up
hsa-miR-6068	4.99E-03	5.34	Up	6.69E-02	3.41	Up
hsa-miR-5193	6.17E-03	5.46	Up	4.19E-02	5.02	Up
hsa-miR-3960	1.04E-02	3.33	Up	5.15E-02	2.47	Up
hsa-miR-4657	1.34E-02	4.55	Up	2.29E-01	2.31	Up
hsa-miR-6089	1.34E-02	3.02	Up	2.32E-01	1.89	Up
hsa-miR-4730	1.34E-02	12.67	Up	6.33E-02	10.01	Up
hsa-miR-4537	1.60E-02	5.35	Up	1.15E-01	3.52	Up
hsa-miR-486-5p	1.96E-02	2.65	Up	2.56E-03	3.74	Up
hsa-miR-106b-3p	2.01E-02	2.38	Up	2.48E-03	3.16	Up
hsa-miR-6825-3p	2.01E-02	4.18	Up	2.36E-01	2.28	Up
hsa-miR-3615	5.06E-02	1.87	Up	2.56E-03	3.09	Up
hsa-miR-106b-5p	7.44E-02	1.93	Up	4.64E-03	3.12	Up
hsa-miR-25-3p	7.44E-02	2.03	Up	5.68E-03	3.46	Up
hsa-miR-484	1.00E-01	1.96	Up	3.10E-04	5.34	Up
hsa-miR-20b-5p	1.12E-01	1.91	Up	8.35E-04	3.57	Up
hsa-miR-20a-5p	4.17E-01	1.34	Up	1.02E-03	3.19	Up

Supplementary Table 2: Logistic regression model of classifiers for plasma / serum miRNA combinations in HCC vs cirrhosis patients or healthy controls

			**miRNAs combinations**	**Logistic regression model**
Plasma	HCC vs Cirrhosis	Classifier 1	miR–101–3p + miR–106b–3p + miR–1246	–20.04 + 3.43 x miR–101–3p + 5.69 x miR–1246 + 3.00 x miR–106b–3p
		Classifier 2	miR–101–3p + miR–106b–3p	–3.73 + 1.83 x miR–101–3p + 0.50 x miR–106b–3p
		Classifier 3	miR–101–3p + miR–1246	–7.71 + 1.64 x miR–101–3p + 2.97 x miR–1246
		Classifier 4	miR–106b–3p + miR–1246	–9.33 + 1.35 x miR–106b–3p + 3.80 x miR–1246
Plasma	HCC vs Healthy controls	Classifier 1	miR–101–3p + miR–106b–3p + miR–1246	–61.83 + 10.26 x miR–101–3p + 3.52 x miR–1246 + 20.41 x miR–106b–3p
		Classifier 2	miR–101–3p + miR–106b–3p	–51.75 + 9.83 x miR–101–3p + 20.34 x miR–106b–3p
		Classifier 3	miR–101–3p + miR–1246	–2.43 + 0.48 x miR–101–3p + 1.27 x miR–1246
		Classifier 4	miR–106b–3p + miR–1246	–7.91 + 2.45 x miR–106b–3p + 1.79 x miR–1246
Serum	HCC vsCirrhosis	Classifier	miR–101–3p + miR–106b–3p	–2.49 – 0.26 x miR–101–3p + 2.09 x miR–106b–3p

Supplementary Table 3: Association of clinic-pathological characteristics of HCC patients (cohort 3) with levels of circulating miRNAs

		**miR-101-3p**		***p* value**	**miR-1246**		***p* value**	**miR-106b-3p**		***p* value**
**HCC Plasma level cutoff (copies/ul)[a]**		**< 31.17 *n* = 15**	**> 31.17 *n* = 7**		**< 21.67 *n* = 16**	**> 21.67 *n* = 6**		**< 5.03 *n* = 12**	**> 5.03 *n* = 10**	
**Sex**	Male	*n* = 11	*n* = 5	1.00E + 00	*n* = 13	*n* = 3	2.00E-01	*n* = 8	*n* = 8	1.00E + 00
	Female	*n* = 4	*n* = 2		*n* = 3	*n* = 3		*n* = 4	*n* = 2	
**Age(years)**	< 60	*n* = 1	*n* = 1	1.00E + 00	*n* = 0	*n* = 2	6.00E-02	*n* = 1	*n* = 1	1.00E + 00
	≥ 60	*n* = 14	*n* = 6		*n* = 16	*n* = 4		*n* = 11	*n* = 9	
**Etiology**	HBV	*n* = 6	*n* = 1	1.00E-01	*n* = 6	*n* = 1	3.00E-01	*n* = 6	*n* = 1	3.00E-01
	HCV	*n* = 4	*n* = 5		*n* = 5	*n* = 4		*n* = 5	*n* = 4	
**Grading**	G1	*n* = 4	*n* = 1	6.00E-01	*n* = 3	*n* = 2	5.00E-01	*n* = 2	*n* = 3	6.00E-01
	G2-G4	*n* = 11	*n* = 6		*n* = 13	*n* = 4		*n* = 10	*n* = 7	
**AFP (ng/µl)**	< 20	*n* = 3	*n* = 3	3.00E-01	*n* = 4	*n* = 2	1.00E + 00	*n* = 4	*n* = 4	1.00E-01
	≥ 20	*n* = 12	*n* = 4		*n* = 12	*n* = 4		*n* = 8	*n* = 6	
**Nodule size(cm)**	< 3	*n* = 4	*n* = 3	6.00E-01	*n* = 5	*n* = 2	1.00E + 00	*n* = 4	*n* = 3	1.00E + 00
	≥ 3	*n* = 11	*n* = 4		*n* = 11	*n* = 4		*n* = 8	*n* = 7	

[a]The mean value of plasma copies / ul was chosen as cutoff value.
